# Nickel Hollow Spheres Concatenated by Nitrogen‐Doped Carbon Fibers for Enhancing Electrochemical Kinetics of Sodium–Sulfur Batteries

**DOI:** 10.1002/advs.201902617

**Published:** 2019-12-23

**Authors:** Bingshu Guo, Wenyan Du, Tingting Yang, Jianhua Deng, Dingyu Liu, Yuruo Qi, Jian Jiang, Shu‐Juan Bao, Maowen Xu

**Affiliations:** ^1^ Key Laboratory of Luminescent and Real‐Time Analytical Chemistry (Southwest University) Ministry of Education School of Materials and Energy Southwest University Chongqing 400715 P. R. China

**Keywords:** effective catalysis, freestanding cathodes, nickel hollow spheres, reaction kinetics, sodium–sulfur batteries

## Abstract

The high energy density of room temperature (RT) sodium–sulfur batteries (Na‐S) usually rely on the efficient conversion of polysulfide to sodium sulfide during discharging and sulfur recovery during charging, which is the rate‐determining step in the electrochemical reaction process of Na‐S batteries. In this work, a 3D network (Ni‐NCFs) host composed by nitrogen‐doped carbon fibers (NCFs) and Ni hollow spheres is synthesized by electrospinning. In this novel design, each Ni hollow unit not only can buffer the volume fluctuation of S during cycling, but also can improve the conductivity of the cathode along the carbon fibers. Meanwhile, the result reveals that a small amount of Ni is polarized during the sulfur‐loading process forming a polar Ni—S bond. Furthermore, combining with the nitrogen‐doped carbon fibers, the Ni‐NCFs composite can effectively adsorb soluble polysulfide intermediate, which further facilitates the catalysis of the Ni unit for the redox of sodium polysulfide. In addition, the in situ Raman is employed to supervise the variation of polysulfide during the charging and discharging process. As expected, the freestanding S@Ni‐NCFs cathode exhibits outstanding rate capability and excellent cycle performance.

## Graphical abstract

A network‐like Ni‐NCFs composite (nitrogen‐doped carbon fibers implanted with Ni) with excellent conductivity and catalysis is synthesized as host for Na‐S batteries. Among it, the Ni hollow units both can buffer the volume fluctuation of S and accelerate the redox kinetics of Na_2_Sx. Benefiting from the structural and compositional advantages, as‐assembled batteries show outstanding rate capability and improved cycling ability.

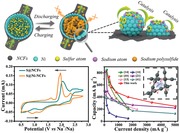

## 1 Introduction

In recent years, the fast growing of electric vehicle technology and human's ambition for efficacious utilization of the clean renewable resources offer a great opportunity for the advancement of electrochemical energy storage technologies (ESSs), while simultaneously bringing several challenges. Since the first commercialization of the LiCoO_2_//carbon cell in 1991, lithium‐ion batteries (LIBs) still dominate the portable electronic market due to the excellent reliability and mature techniques for battery assembly.^1^, ^2^ However, considering the low reserves abundance and uneven distribution of lithium resources, as well as the limited energy density, the LIBs always fall short in the large‐scale stationary energy storage systems, such as in electrical grids.^3^, ^4^ In this regard, sodium–sulfur (Na‐S) batteries automatically stand out due to their overwhelming advantages in terms of remarkable theoretical energy density and exceptionally low cost for both anode (metallic sodium) and cathode (sulfur).^5^, ^6^ Moreover, sodium, as an alternative to lithium, is available in all regions over the world, further driving the rapid development of Na‐S batteries.^7^

The development of the conventional Na‐S battery can be traced to the 1960s. Generally, its actual operating temperature at around 270 to 350 °C with molten Na/S as electrodes and a solid beta‐alumina as electrolyte is now commercially available.^8^ Although such high temperature (HT) Na‐S batteries show satisfactory thermal stability and high efficiency with long cycle life, the strong corrosivity of molten polysulfides at HT always results in serious safety issues, high maintenance cost, and low reliability.^9^ Therefore, to realize the large‐scale application of Na‐S batteries and overcome these challenges, room temperature (RT) Na‐S batteries have been proposed since 2006.^10^ Unfortunately, the operation of RT Na‐S batteries is facing some fundamental problems that remain to be addressed. First, the low electronic conductivity and huge volume expansion (160%) of S are the main obstacles, which will lead to low utilization of active species and destructive structural collapse of electrode. Second, the shuttling of soluble sodium polysulfide (Na_2_S*_x_*, 4 ≤ *x* ≤ 8) intermediate is another question, which will eventually undergoes redox reactions to form lower‐order polysulfides (Na_2_S_2_ and Na_2_S) on the anode surface, finally leading to a significant reduction in capacity. In addition, Na has a lower reactivity with S than that of Li due to its larger ion radius.^11^, ^12^

Against this background, for an anticipant cathode of Na‐S batteries, the sulfur host needs to meet the following requirements generally to alleviate the performance deterioration: first, the host should possess desirable electronic conductivity to counteract that of S; besides, abundant pore structure or hollow structure is needed to buffer the volume expansion of S and physically restrict the shuttle of polysulfide; finally, as proved by reported literatures that the polar material with intrinsic sulfiphilic property is necessary because of the excellent chemical adsorption for soluble polysulfide. Therefore, several homologous hosts have been explored to improve the electrochemical performances of RT Na‐S and Li‐S batteries, such as heteroatom‐doped porous carbon,^13^, ^14^, ^15^ metal oxides (TiO_2,_ MnO_2_, Fe_3_O_4_),^16^, ^17^, ^18^ and metal sulfides (FeS_2_, Co_9_S_8_).^19^, ^20^ But anyway, the adsorption of the polar surface for polysulfide is insufficient, and thus the shuttle effect of sodium polysulfide still exists. Recently, according to Dou's group report that the transition metal atoms can speed up the redox kinetics of the conversion from long‐chain to short‐chain polysulfide and ultimately to Na_2_S.^21^, ^22^ This conception can not only rapidly eliminate the soluble sodium polysulfide and improve the capacity of the battery, but also enhance the overall conductivity of the cathode. Therefore, finding suitable hosts with the transition metal atoms as catalytic centers along with physical/chemical adsorption is a promising direction for developing superior Na‐S batteries.

**Figure 1:**
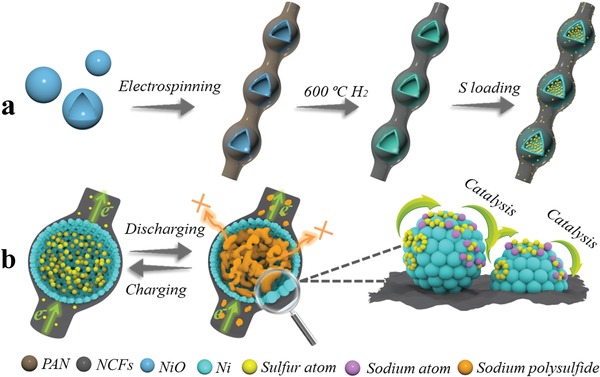
a) Schematic illustration for the preparation of S@Ni‐NCFs composite and b) its advantages during charging and discharging processes.

In this work, we designed and synthesized a 3D host consisting of carbon fiber concatenated nickel hollow sphere for accelerating the electrochemical kinetics of RT Na‐S batteries. The typical preparation process is schematically depicted in **Figure** **1**a. NiO hollow spheres with a uniform diameter of about 850–950 nm are first achieved by calcining Ni‐based MOF at 500 °C in air atmosphere, and then they are incorporated into polyacrylonitrile (PAN) fibers by electrostatic spinning. After pre‐oxidation in the air at 280 °C,^23^, ^24^ the NiO‐PAN precursors are sintered at 600 °C in Ar/H_2_ atmosphere, during which the NiO is reduced to Ni while the PAN is carbonized to carbon fiber. Finally, after a vacuum heating process, the obtained Ni‐NCFs are employed as substrate for loading S to get S@Ni‐NFCs composite.

In our proposed unique structure (Figure 1b), each nickel unit displays a hollow structure, which can accommodate the volumetric expansion of S during cycles. Meanwhile, the nickel atoms also exhibit a significant catalytic effect in the conversion of polysulfide to accelerate the reaction kinetics of liquid intermediates, finally alleviating the shuttle effect to some extent. In addition, the cross‐linked carbon fibers with wrapped nickel hollow spheres allow electrons flow freely and unimpeded in different directions. And thus the electrode harvests excellent electron conductivity. Importantly, the S@Ni‐NCFs can be directly used as self‐supporting electrodes without adding any binders and conductive additives, which would avoid the complex electrode‐preparation process and increase the overall mass ratio of active species in the battery system. Due to the above advantages, the as‐assembled Na‐S batteries show excellent rate capability and improved cycle performance when compared with the composite of pristine nitrogen‐doped carbon fiber and sulfur (S@NCFs).

## 2 Results and Discussion

The morphology of the Ni‐MOF precursor from the coordination of 1,3,5‐benzenetricarboxylic acid (H_3_BTC) and Ni^2+^ is first characterized by field emission scanning electron microscopy (FESEM) and transmission electron microscopy (TEM) as shown in Figure S1a–c in the Supporting Information. They are presented as intact and solid spheres with a smooth surface and the diameter of 1.3–1.5 µm. The X‐ray diffraction (XRD) pattern as exhibited in Figure S1d in the Supporting Information agrees well with the reported literatures.^25^ After calcination in air at 500 °C, the organic components in the precursor escape while spontaneously the metal ingredients convert into hollow structure with reduced size (0.8–0.95 µm) and rough surface, which can be confirmed by the FESEM images and the energy‐dispersive X‐ray spectroscopy (EDS) elemental mapping in Figure S2a–c in the Supporting Information. The EDS elemental mapping images in the inset of Figure S2c in the Supporting Information reveal the homogenous distributions of Ni and O without any signal from C element. The quantitative analysis (Figure S2c, Supporting Information) further indicates that the atomic ratio of Ni and O is about 1:1. The TEM image in Figure S2d in the Supporting Information confirms the hollow feature of NiO, which is coincided with the FESEM characterization. Figure S2e in the Supporting Information further reveals that the hollow structure is composed of a large number of NiO nano‐particles. The XRD pattern in Figure S3a in the Supporting Information demonstrates that all the diffraction peaks are well corresponded to pure NiO (JCPDS card No. 44‐1159), without any impurity and intermediate phases.^26^ Moreover, as shown in Figure S3b in the Supporting Information, the Brunauer–Emmett–Teller (BET) specific surface area and pore volume of NiO are 20.2 m^2^ g^−1^ and 0.15 cm^3^ g^−1^, respectively. Along with the pore diameter distribution curve in the inset of Figure S3b in the Supporting Information, it can be seen that the macropores and mesopores mainly exist in NiO hollow spheres.

**Figure 2:**
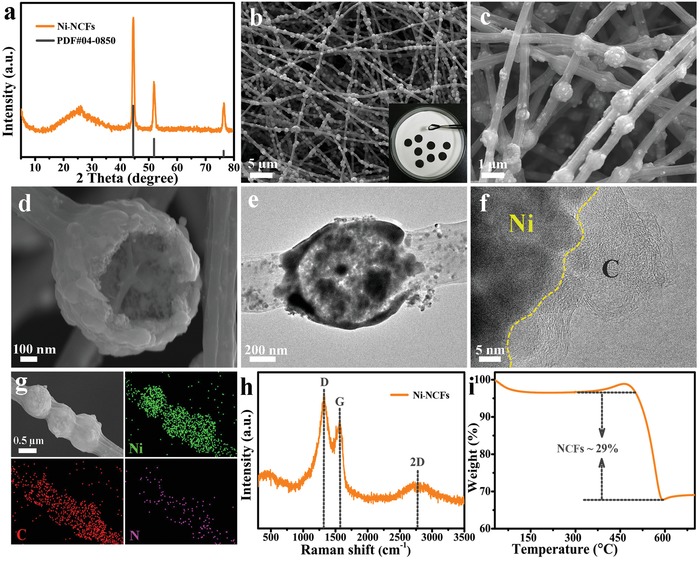
Characterization of the Ni‐NCFs composite. a) XRD pattern, b–d) FESEM, e,f) TEM, g) EDS elemental mapping images, h) Raman spectrum, and i) TGA curve in the air atmosphere.

Subsequently, the NiO hollow spheres are mixed with PAN in dimethylformamide solution to fabricate the NiO‐PAN interconnected frameworks by electrospinning. As shown in Figure S4a,b in the Supporting Information, each NiO sphere is connected in series and well wrapped by PAN fibers forming a 3D network. According to the XRD pattern in **Figure** **2**a, after heat treatment of NiO‐PAN in H_2_ at 600 °C, several sharp diffraction peaks appeared at *2θ* of 44.5°, 51.8°, and 76.6° can be indexed to the (1 1 1), (2 0 0) and (2 2 0) lattice planes of metallic Ni (JCPDS No. 04‐0850), respectively, while a broad diffraction peak located at *2θ* = 26° is assigned to (0 0 2), the plane of partially graphitized carbon.^27^, ^28^ It is declared that the NiO is thoroughly reduced to metallic Ni while the PAN is carbonized to carbon fiber. Simultaneously, the FESEM images of Ni‐NCFs (Figure 2b,c) reflect that the 3D conductive network structure is perfectly maintained after carbonization, while the diameter of the carbon fiber (≈400 nm) becomes smaller than that of the precursor (≈500 nm). Besides, the photo of the cropped Ni‐NCFs disks (illustration of Figure 2b) reveals the excellent flexibility and mechanical property of the as‐obtained self‐standing electrode. From the view of a broken sphere in Figure 2d, it can be clearly observed that Ni unit remains as a hollow structure, which is also confirmed by the TEM image in Figure 2e. The high‐resolution TEM (HRTEM) image of Ni‐NCFs (Figure 2f) reveals that the Ni nanoparticles are well embedded in the carbon matrix. As shown in Figure 2g, the EDS elemental mapping images disclose the homogenous distribution of Ni, C, and N, indicating the carbon fibers are doped with nitrogen as well, which is helpful to adjust the nonpolar property of the carbon matrix. To further investigate the carbon component, Raman characterization is carried out and presented as Figure 2h. The D‐band and G‐band at about 1318 and 1565 cm^−1^ are related to the defective and graphitic carbon, respectively, while the 2 D band at around 2780 cm^−1^ is associated with highly crystalline carbon material.^29^ The high *I*_D_/*I*_G_ value and wide 2 D band suggest a low graphitization degree of the carbon in Ni‐NCFs composite, which is beneficial for the insertion of sodium ions during cycling. The carbon content in the Ni‐NCFs composite is calculated to be about 29% based on the thermogravimetric analysis (TGA; Figure 2i). Additionally, the N_2_ adsorption/desorption isotherms and pore size distribution curve of Ni‐NCFs shown in Figure S5 in the Supporting Information display the BET specific surface area of 35.6 m^2^ g^−1^ and pore volume of 0.09 cm^3^ g^−1^ with mesopores and macropores. In contrast, the pure nitrogen‐doped carbon fibers (NCFs; Figures S6 and S7, Supporting Information) present a lower BET specific surface area and pore volume (22.7 m^2^ g^−1^ and 0.13 cm^3^ g^−1^). It is observed that the specific surface area of Ni‐NCFs is larger and the pore structure is more abundant, which may result from the plentiful gaps and spaces offered by joints of hollow spheres and carbon fibers.

**Figure 3:**
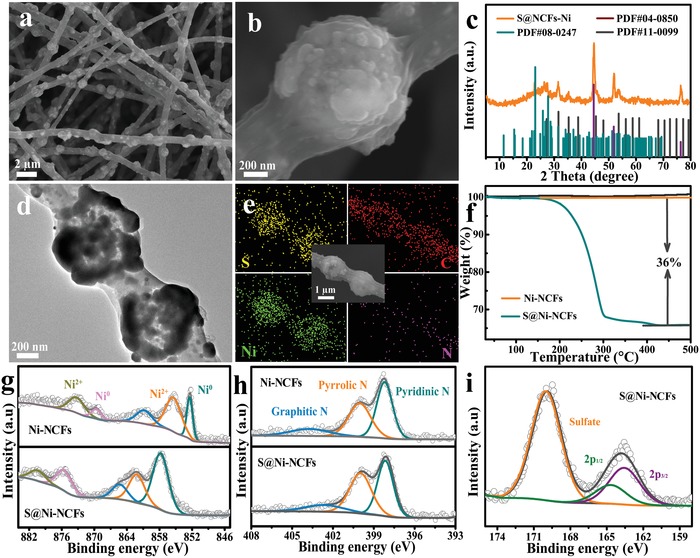
Characterization of the S@Ni‐NCFs composite. a,b) FESEM, c) XRD pattern, d) TEM, e) EDS elemental mapping images, and f) TGA curve, g,h) high‐resolution XPS spectra of Ni 2p and N 1s from Ni‐NCFs and S@Ni‐NCFs, and i) high‐resolution XPS spectra of S 2p from S@Ni‐NCFs composite.

The S@Ni‐NCFs cathode is prepared by melting diffusion of S into the Ni hollow spheres and N‐doped carbon fibers of Ni‐NCFs at 155 °C. **Figure** **3**a,b displays the FESEM images of the composite, it can be seen that the morphologies of the products after S steaming remain basically unchanged and there is almost no excess S block attached on outside surface of the material. From the XRD pattern (Figure 3c), in addition to the characteristic diffraction peaks of Ni, the diffraction peaks of S_8_ and NiS_2_ can also be observed. The TEM image in Figure 3d shows that the inner wall of the Ni hollow sphere becomes darker, further indicating the successful encapsulation of S into the cavity along the internal accumulation of inner shell. Moreover, the EDS elemental mapping images (Figure 3e) clearly affirm that S is well‐embedded in Ni‐NCFs composite. In Figure 3f, the TGA curve displays a prominent weight stage, which is caused by the sublimation of S. The result indicates that the S content in S@Ni‐NCFs is about 36%. In addition, as shown in Figure S5 in the Supporting Information, the specific surface area and pore volume of S@Ni‐NCFs (13.4 m^2^ g^−1^ and 0.03 cm^3^ g^−1^) decrease obviously when compared with the pristine Ni‐NCFs (35.6 m^2^ g^−1^ and 0.09 cm^3^ g^−1^). Above results suggest that the S is successful diffused into the Ni‐NCFs. Meanwhile, the existence of some mesopores after the loading of S may serve as an accommodation for the volume expansion of S during cycles.

To further compare the superficial bonding information of Ni‐NCFs and S@Ni‐NCFs, the X‐ray photoelectron spectroscopy (XPS) measurement is performed. As shown in Figure 3g, before loading of S, the peaks at bonding energies of ≈873.2 eV (Ni 2p_3/2_) and 855.3 eV (Ni 2p_1/2_) are assigned to metallic Ni, while the peaks at binding energies of ≈873.2 eV (Ni 2p_3/2_) and 869.5 eV (Ni 2p_1/2_) correspond to the Ni^2+^ due to the partial oxidation of Ni on the surface of the product. In addition, a peak at the binding energy of 860.7 eV is derived from the satellite peak of Ni.^30^ For the N 1s spectrum, three types of N species can be detected in the Ni‐NCFs composite (Figure 3h), including pyridinic N, pyrrolic N, and graphitic N at binding energies of 398.2, 400.0, and 403.7 eV, respectively.^15^ After loading of S, the shape and position of N 1s peaks remain basically unchanged, while all peaks in the Ni 2p spectrum move to higher binding energies. This phenomenon may suggest the formation of the Ni—S bonds between metallic Ni and S. The S 2p spectrum in Figure 3i is well supported for above conjecture. The dual peaks appeared at 163.5 and 164.9 eV with a splitting energy of ≈1.4 eV are assigned to S 2p_3/2_ and S 2p_1/2_, respectively. Compared with pure S (at ≈163.9 and ≈165.1 eV), the binding energies for both S 2p_3/2_ and S 2p_1/2_ decrease, which is well matched with typical S 2p spectrum of Ni—S reported by Niu et al. and our previous report.^31^, ^32^ These facts affirm the presence of a few polar Ni—S bonds on the surface of S@Ni‐NCFs composite, which is helpful for improving the electrochemical properties of the Na‐S batteries.

**Figure 4:**
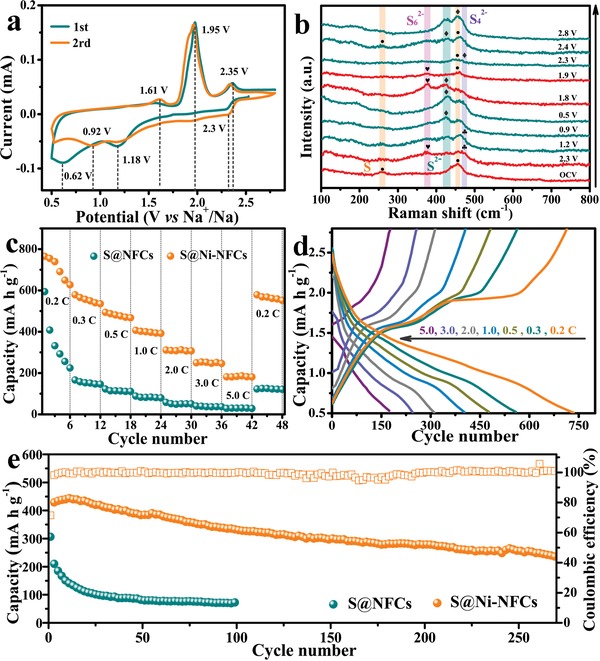
Electrochemical performance characterization. a) CV curves at the scan rate of 0.1 mV s^−1^, b) in situ Raman analysis of S@Ni‐NCFs electrode at different charge/discharge stages, c) rate capabilities, d) discharge/charge curves, and e) cycling performances at 1.0 C of S@Ni‐NCFs and S@NCFs.

The as‐obtained S@Ni‐NCFs freestanding electrode is used as cathode against sodium to evaluate its electrochemical properties and energy storage mechanism in Na‐S batteries. The cyclic voltammetry (CV) and in situ Raman are measured to trace the changes of sulfur species during the discharge/charge process. As shown in **Figure** **4**a, the CV curve shows three prominent peaks at near 2.30, 1.18, and 0.62 V during the first cathodic scan. Among them, the peak located at 2.30 V is originated from the conversion of solid S_8_ to soluble long‐chain polysulfide (Na_2_S*_n_*, *n* = 4–8), while the peak at around 1.18 V results from the subsequent solidification of Na_2_S*_n_* to short‐chain polysulfide (Na_2_S*_x_*, *x* = 1–3) and the formation of solid electrolyte interphase film. The broad peak at around 0.62 V may stem from the sodiation of the small quantity of NiS_2_.^33^, ^34^ Meanwhile, as exhibited by the in situ Raman spectrum in Figure 4b, when the battery is in static state, the characteristic peaks of S_8_ emerge at 259 and 456 cm^−1^.^35^ After discharging to 2.3 V, the peak intensity of S_8_ becomes weak gradually and other two new peaks originated from Na_2_S_6_ and Na_2_S_4_ appear at about 374 and 473 cm^−1^, respectively. When the discharge process continues to 1.2 V, the peak of Na_2_S at about 428 cm^−1^ is observed and its strength increases continuously until fully discharged to 0.5 V.^36^ In should be noted that the peaks of S_8_ always exist throughout the whole discharge process, which suggests that the S_8_ is not completely reduced due to its poor electronic conductivity and limited reactivity. Afterward, the subsequent charge as an opposite process shows three anodic peaks at near 1.65, 1.95, and 2.35 V in the CV curves of Figure 4a. The first weak anodic peak at 1.65 V corresponds to the reversible oxidation process of Ni to NiS_2_, while the other two peaks are ascribed to the oxidation of Na_2_S to short‐chain Na_2_S*_x_* (1.95 V) and the successive conversion of Na_2_S*_x_* to long‐chain sodium polysulfide and finally to S (2.35 V).^34^ Accordingly, when the cell is charged to 1.8 V, the peak at 375 cm^−1^ emerged in the Raman spectrum indicates the formation of Na_2_S_6_.^37^ After the cell is further charged back to 2.8 V, the peaks of both S and deposited Na_2_S can be detected, revealing that the reaction is partially reversible. However, during the subsequent scan, the cathodic and anodic peaks shift toward higher and lower potential, respectively.

Figure 4c shows the comparison of the rate capability of S@NCFs and S@Ni‐NCFs cathodes with the same sulfur content (Figure S8, Supporting Information). As displayed, with the increase of the current density from 0.2 C to 5.0 C (1 C = 1675 mAh g^−1^), the specific capacity of the S@NCFs composite decreases rapidly from 593 to 31 mAh g^−1^. By contrast, the S@Ni‐NCFs cathode can deliver much higher average reversible specific capacities of about 738.7, 565.6, 481.1, 401.9, 311.1, 249.8, and 181.7 mAh g^−1^ at 0.2 C, 0.3 C, 0.5 C, 1.0 C, 2.0 C, 3.0 C, and 5.0 C, respectively. After that, when the current density is switched back to 0.2 C, the capacity can recover to 578.5 mAh g^−1^ with the capacity retention of ≈78.3%, exhibiting an improved rate performance. The typical galvanostatic discharge/charge profiles of S@Ni‐NCFs at different current densities are displayed in Figure 4d. The flat voltage plateaus appeared on the discharging and charging curves are consistent well with the CV curves, which reveals that the capacity is contributed by both S and NiS_2_, simultaneously. To investigate the possible sodium storage performance of NiS_2_ in the S@Ni‐NCFs composite, the S is removed intentionally by CS_2_ to obtain Ni‐NCFs/S composite. As shown by the HRTEM image of Ni‐NCFs/S (Figure S9, Supporting Information), the lattice fringes with interplanar spacing of 0.20 and 0.23 nm exist simultaneously, which are consisted with (111) and (211) planes of Ni and NiS_2_, respectively. Besides, the fast Fourier transform image in the illustration of Figure S9 in the Supporting Information belongs to the (211) plane of NiS_2_ as well, further confirming the presence of NiS_2_ in Ni‐NCFs/S composite. Figure S10 in the Supporting Information displays the electrochemical performance of Ni‐NCFs/S and Ni‐NCFs electrode. After 200 cycles at 0.5 C, the capacity of Ni‐NCFs/S electrode rapidly decays from 103.0 to 31.8 mAh g^−1^, while the capacity of Ni‐NCFs electrode remains at about 16 mAh g^−1^. Above results demonstrate that the capacity contribution from NiS_2_ is negligible even though NiS_2_ does exist in S@Ni‐NCFs composite, but it is not contributed much capacity during charge and discharge processes due to the low content. In addition, the S@Ni‐NCFs electrode presents superior cycling stability than that of S@NCFs as shown in Figure 4e. The initial capacity of S@Ni‐NCFs cathode at 1.0 C is ≈431 mAh g^−1^, which is much higher than that of S@NCFs (only ≈306 mAh g^−1^). After 270 cycles, the capacity of S@Ni‐NCFs can retain ≈233 mAh g^−1^ along with an attenuation rate of 0.17% per cycle and the Coulombic efficiency keeps over 90%. In contrast, the capacity of S@NCFs drops quickly to 71 mAh g^−1^ after 100 cycles under the same condition with attenuation rate as high as 0.77% per cycle. The remarkable difference in performance is mainly caused by the distinction of their interfacial electrochemical kinetics, which is closely related with chemisorption ability and electronic conductivity of hosts as reported by Zhang et al.^38^ On the one hand, due to the doped‐nitrogen atoms and polar Ni—S bond in the Ni‐NCFs/S electrode, the adsorption and interfacial affinity with polar polysulfide are superior than that of pure carbon fibers (Figure S11, Supporting Information). On the other hand, as shown in Figure S12a and Table S1 in the Supporting Information, the S@Ni‐NCFs displays a smaller charge transfer resistance than the S@NCFs, further demonstrating the favorable electronic conductivity of S@Ni‐NCFs. Besides, S@Ni‐NCFs electrode also reveals a faster sodium ions diffusion rate according to the fitted slope by electrochemical impedance spectroscopy (EIS) spectra (Figure S12b, the details are displayed in the Supporting Information).

To explore the reasons of capacity decay, the FESEM images of S@NCFs and S@Ni‐NCFs after cycling are shown in Figure S13 in the Supporting Information. There is a deposited layer appears on the surface of both cathodes, which should be nonconductive Na_2_S. Obviously, the deposition amount on the surface of S@Ni‐NCFs is much less than that of S@NCFs due to the catalytic action of Ni. In addition, only a small quantity of nickel hollow spheres in the S@Ni‐NCFs electrode still remains intact. Thus, it can be concluded that the attenuation of the capacity mainly originates from the collapse of electrode microstructure and the loss of active substances caused by the deposition of Na_2_S. Moreover, the electrochemical performance of the S@Ni‐NCFs electrode with a relatively high sulfur loading of 45% is satisfactory as well (Figure S14, Supporting Information). The discharge capacity at 0.2 C is as high as 703 mAh g^−1^ (almost equals to that of electrode with 36% S loading), even though the rapid capacity decays at high current rate and long‐term cycling because of the worse conductivity.

**Figure 5:**
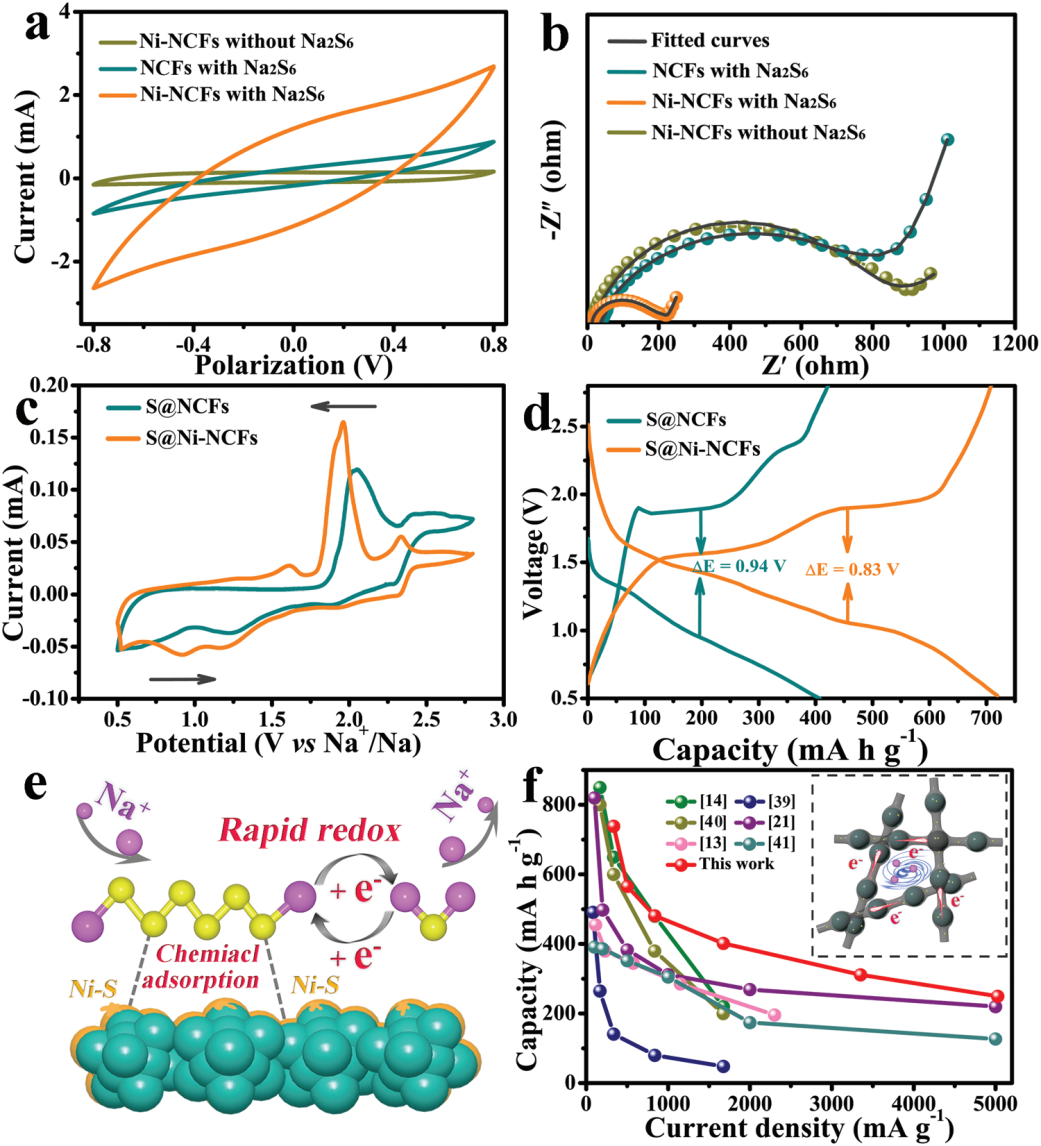
Catalytic and adsorption characterization. a) Polarization curve and b) EIS of Na_2_S_6_ symmetric cells, c) comparison of CV and d) discharge/charge curves of S@Ni‐NCFs and S@NCFs electrodes. e) Schematic diagram of S@Ni‐NCFs about the polysulfide adsorption and conversion processes on the surface of metallic Ni, f) rate contrast between the published literatures^39^, ^40^, ^41^ and the S@Ni‐NCFs electrode developed in this work.

To verify the above claim, the catalytic effect of Ni‐NCFs substrate on polysulfide transformation is indirectly probed by the CV of symmetric cell with Na_2_S_6_ electrolyte. As shown in **Figure** **5**a, ignoring the cell without adding Na_2_S_6_, it can be clearly observed that the Ni‐NCFs electrode presents a larger current response than that of the pristine NCFs electrode, demonstrating the helpful effect of metallic Ni to accelerate the electrochemical reactions of sodium polysulfides. Besides, as shown in Figure 5b and Table S1 in the Supporting Information, the charge transfer resistance of the Ni‐NCFs electrode is remarkably reduced when compared with pure NCFs electrode. Since the sodium‐free condition, this result may be derived from the enhanced electronic conductivity and interfacial affinity between the substrate and polysulfide. In contrast, the relatively poor interfacial affinity and electronic conductivity between NCFs and polysulfide always lead to an extra surface diffusion step, which impedes superficial conversion and subsequent nucleation of Na_2_S, thus slowing down reaction kinetics. The accelerated dynamics of the Ni‐NCFs electrode can also be directly reflected by the CV and charge/discharge curves of as‐assembled Na‐S batteries as displayed in Figure 5c,d, where the S@Ni‐NCFs cathode shows smaller peak difference and polarization voltage, respectively. As the above analyses, the conductive Ni‐NCFs host materials can accelerate electrochemical reaction kinetics by catalyzing transformation of liquid polysulfide to solid Na_2_S, whereas pure NCFs cannot. Therefore, taking consideration of the experimental data and the theoretical analysis, the adsorption and conversion process of sodium polysulfide on the surface of the Ni‐NCFs can be briefly depicted by Figure 5e. First, the polar bonds on the surface of Ni‐NCFs material can anchor the mobile polysulfide through effective chemisorption, and then a rapid redox can take place on the surface of metallic Ni because of the excellent electronic conductivity and catalytic feature. Benefiting from the structural merits, adsorption and catalysis of S@Ni‐NCFs electrode (Figure 5f), the as‐assembled RT Na‐S battery shows outstanding rate capability when compared to previous literatures. Finally, Table S2 in the Supporting Information exhibits a comprehensive comparison between the performance of this work and other reports.

## 3 Conclusion

In summary, a flexible and freestanding S@Ni‐NCFs cathode is successfully prepared by electrostatic spinning, subsequent carbonization, and sulfur‐loading process. Among it, the Ni hollow spheres are connected in series along with carbon fibers, which can not only contribute to the transportation of electrons, but also effectively accommodate the volume expansion of active species. Meanwhile, the catalytic effect of Ni units on the conversion of polysulfide is well demonstrated by comparing the current response and potential polarization of S@Ni‐NCFs and S@NCFs cathodes. Therefore, along with the Ni—S polar bonds and nitrogen heteroatoms, the S@Ni‐NCFs composite can inhibit the shuttle of polysulfide. As a consequence, the RT Na‐S batteries assembled with S@Ni‐NCFs deliver a high capacity of 738.7 mAh g^−1^ at 0.2 C and remain 249.8 mAh g^−1^ at 2.0 C. After 270 cycles at 1.0 C, the capacity decay rate of S@Ni‐NCFs cathode is only about 0.17% per cycle, which is much lower than that of S@NCFs (≈0.77%). The excellent performance suggests that improving the electronic conductivity and making the best of catalysis are two important principles for the design of cathode materials of Na‐S batteries, which indicates the direction of our future efforts.

## Conflict of Interest

The authors declare no conflict of interest.

## Supporting information

Supporting Information
