# Supplementary material for: Nickel Hollow Spheres Concatenated by Nitrogen‐Doped Carbon Fibers for Enhancing Electrochemical Kinetics of Sodium–Sulfur Batteries
Source: Adv Sci (Weinh). 2019 Dec 23;7(4):1902617. doi: 10.1002/advs.201902617 (PMC7029643; doi:10.1002/advs.201902617)
Supplement: Supplementary file 1 — Supporting Information [file ADVS-7-1902617-s001.pdf]

Copyright WILEY-VCH Verlag GmbH & Co. KGaA, 69469 Weinheim, Germany, 2019.

Supporting Information

for *Adv. Sci.*, DOI: 10.1002/advs.201902617

Nickel Hollow Spheres Concatenated by Nitrogen-Doped Carbon Fibers for Enhancing Electrochemical Kinetics of Sodium–Sulfur Batteries

Bingshu Guo, Wenyan Du, Tingting Yang, Jianhua Deng, Dingyu Liu, Yuruo Qi, Jian Jiang, Shu-Juan Bao, and Maowen Xu**

Copyright WILEY-VCH Verlag GmbH & Co. KGaA, 69469 Weinheim, Germany, 2019.

Supporting Information

Nickel hollow spheres concatenated by nitrogen-doped carbon fibers for enhancing electrochemical kinetics of sodium-sulfur batteries

Bingshu Guo, Wenyan Du, Tingting Yang, Jianhua Deng, Dingyu Liu, Yuruo Qi, Jian Jiang, Shu-Juan Bao, and Maowen Xu**

Experimental Section

All chemical reagents are analytically pure and do not require further purification.

Synthesis of NiO hollow spheres. The Ni-MOF precursors were first prepared by our previously reported method.[1] Specifically, 2.0 mmol $Ni(NO_3)_2$•$6H_2O$ and 2.0 mmol 1,3,5-benzenetricarboxylic acid (H_3BTC) were mixed and dissolved in 60 mL N,N-dimethylformamide (DMF) solution with continuous stirring for 30 min. Then, the green transparent solution was transferred into a 100 mL Teflon-lined stainless steel autoclave and heated at 150 ºC for 12 h. After cooling down to room temperature, the products were collected by filtration and dried at 60 ºC. Afterwards, the NiO hollow spheres were obtained by annealing of as-prepared Ni-MOF at 500 °C under an air atmosphere for 40 min with a heating rate of 1 °C/min.

Synthesis of Ni-NCFs networks. Firstly, the NiO-PAN was prepared via a facile electrospinning process. Typically, 500 mg NiO hollow spheres were dispersed into 6 mL DMF solution by ultrasonic for 10 min. Then 500 mg PAN (MW = 150000) was added into the dispersion solution under magnetic stirring for 12 h to obtain the precursor solution. Next, the mixture solution was sucked into a 5 mL plastic syringe with a blunt tip needle and started to spinning at 30 kV onto a collector covered with aluminium foil . The flow rate and distance between needle and the receiver were kept 0.0008 mm s^{-1} and 18 cm, respectively. After drying at 60 ºC, the NiO-PAN precursors were first pre-oxidized at 280 ºC and pressed into a small round discs with the diameter about 1.3 cm before pyrolysising at 600 °C under Ar/H_2 atmosphere to achieve Ni-NCFs networks. For contrast, the pure NCFs were prepared through the same process, but without adding NiO hollow sphere.

Synthesis of S@Ni-NCFs composite. Firstly, dissolving a certain amount of sulfur powder into carbon disulfide (CS_2) solution obtained a mixture with a concentration of 1.0 mg mL^{-1}. Then, the S solution was dropped on the Ni-NCFs pieces by pipette and heated at 155 °C for 12 h under Ar atmosphere after evaporation of CS_2. Moreover, the S@NCFs composite could

be prepared in the same way for comparing with the same mass loading of sulfur. Finally, in order to detect the sodium storage property of nickel sulfide in the S@Ni-NCFs composite, sulfur was removed from S@Ni-NCFs by using CS_2 solution. The products were labeled as Ni-NCFs/S.

Preparation of Na_2S_6 solution. 320 mg of S and 156 mg of sodium sulfide (Na_2S) were mixed and added into 10 mL tetraglyme (TEGDME) solvent. Then, the suspension was stirred at 80 °C for 6 h to get a reddish-brown Na_2S_6 solution.

Material Characterization

The morphologies of the samples were investigated by field-emission scanning microscope (FESEM, JSM-7800F, Japan) and transmission electron microscopy (TEM, JEM-2100, Japan). The EDS spectroscopy attached to FESEM was employed to record the elemental distribution. The crystal structures were detected through powder X-ray diffraction (XRD, MAXima-X XRD-7000) with Cu Kα radiation (λ = 1.5406 Å). The sulfur and carbon contents in the prepared composites were determined by Thermogravimetric analyzer (TGA, Q50, USA). The BET specific surface area and pore structure were tested by Brunauer-Emmett-Teller method (BET, Quantachrome Instruments, USA). The Thermo Scientific ESCALAB 250Xi electron spectrometer was applied to collect the X-ray photoelectron spectroscopy (XPS) spectra. In addition, ex-situ Raman was recorded by using Invia Refl (Renishaw, UK) from 100 to 3000 cm^{-1}, while the in-situ Raman was recorded by Lab-RAM HR Evolution (Horiba, EL-CELL in Germany) Raman microscope with a computer controller (CHI 660D).

Assembly and measurement of Na-S batteries

All electrochemical measurements were studied at room temperature by assembling CR2032 coin cells in an argon glove-box. The S@Ni-NCFs and S@NCFs wafers with an area load of about 0.5 ~ 0.7 mg cm^{-2} were directly used as working electrodes without adding any conductive additives or PVDF binder. In this system, the sodium foil was served as both the counter and reference electrode, and the glass fiber membrane (Whatman GF/A) was acted as the separator. Meanwhile, 1 M $NaClO_4$ dissolved in tetraethylene glycol dimethyl ether (TEGDME) was used as the electrolyte and the dosage was 90 μL. After stewing for 8 h, the coin cells started galvanostatically charging and discharging within the voltage ranging from 0.5 to 2.8 V on a Land cycler (1C = 1675 mAh g^{-1}, Wuhan Kingnuo Electronic Co, China). Cyclic voltammograms (CV) curves were performed at a scan rate of 0.1 mV s^{-1} using Arbin Instruments. Electrochemical impedance spectroscopy (EIS) was tested by Zahner electrochemical workstation.

Assembly and measurement of symmetrical batteries

Two identical disk electrodes (Ni-NCFs or NCFs) without sulfur load were both used as anode and cathode for assembling into a standard CR2032 coin cell. Among it, the glass fiber membrane was employed as separator, then 40 μL of Na_2S_6 (0.2 mol L^{-1}) and 50 μL of blank electrolyte (1 M $NaClO_4$ in TEGDME) were added. The CV curves were performed at a scan rate of 50 mV s^{-1} between -0.8 V and 0.8 V, while the EIS were measured on Zahner electrochemical workstation.

Calculated diffusion coefficient of sodium ions (D_{Na+})

The diffusion coefficients of sodium ions (D_{Na+}) in S@Ni-NCFs and S@NCFs electrodes can be calculated according to the following equations:

$$D_{Na+} = D_{Na+} = \left(\frac{2RT}{\sqrt{2}n^2F^2\sigma_w AC}\right)^2 = \frac{2R^2T^2}{n^2F^4\sigma_w{}^2A^2C^2} \quad (1)$$

$$Z' = R + \sigma_w w^{-1/2} \quad (2)$$

In equation (1), R, T, n, F, A, and C represent gas constant, absolute temperature, the number of electrons transferred per mole during oxidation, Faraday constant, effective area of work electrode, and Na+ concentration in cathode material, respectively. While in equation (2), R is a resistance parameter representing the combination of solution resistance and charge transfer resistance. From equation (1) and equation (2), it can be found that the D_{Na+} is only associated with the value of σ, which can be obtained by plotting Z' vs. $\omega^{-1/2}$ and the slop is σ.

Supplementary Figureures

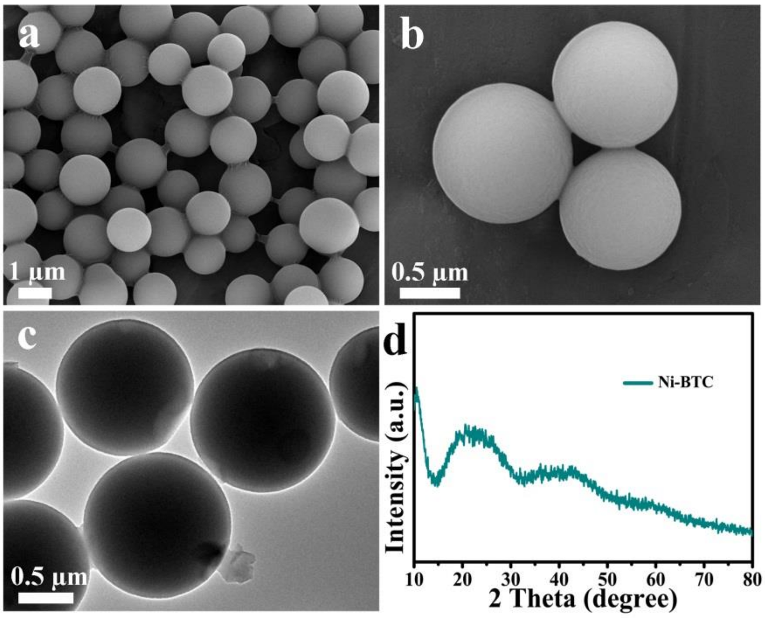

Figure S1. (a-b) FESEM, (c) TEM images and (d) XRD pattern of Ni-MOF

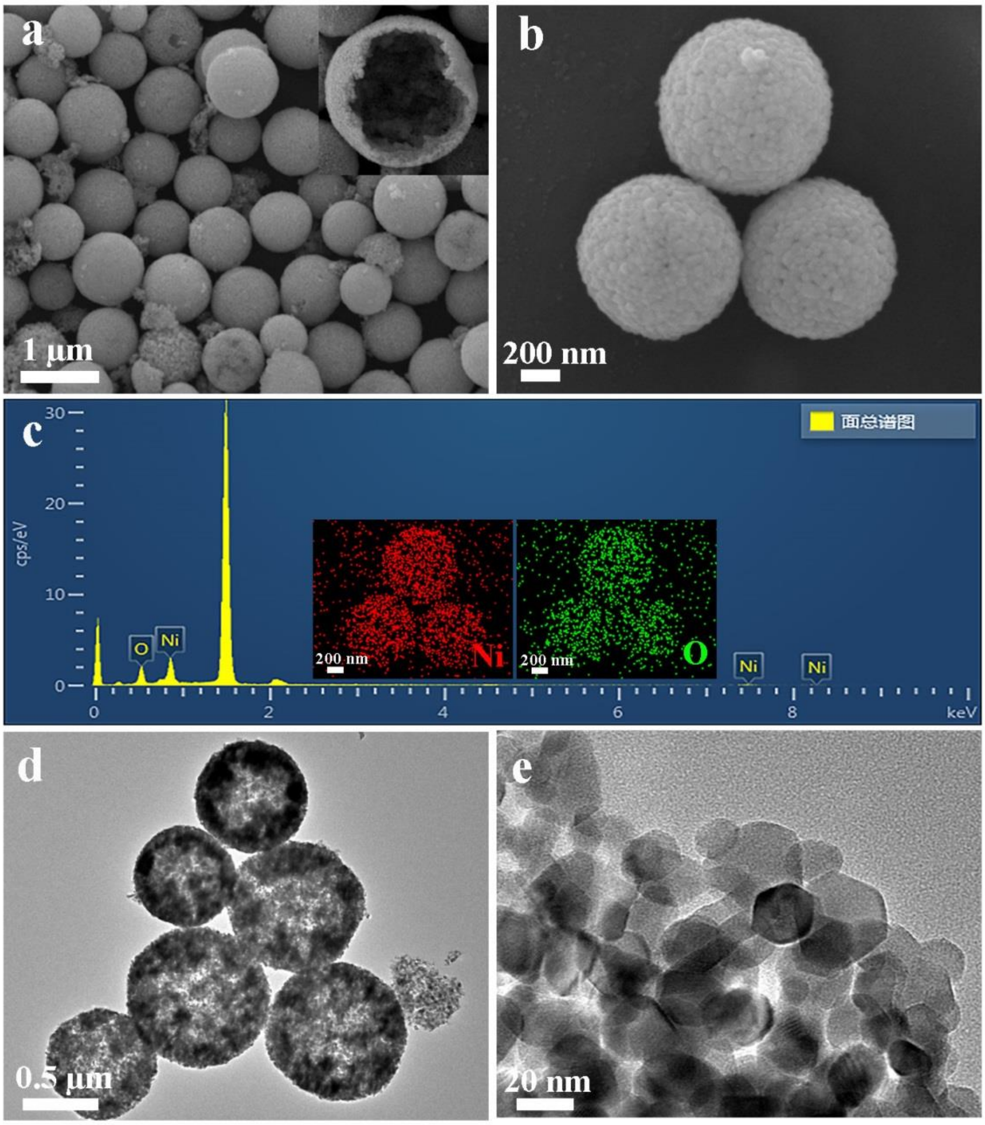

Figure S2. (a-b) FESEM images, (c) EDS spectrum and corresponding EDS mappings of NiO, (e) TEM and (f) HRTEM images of NiO hollow spheres

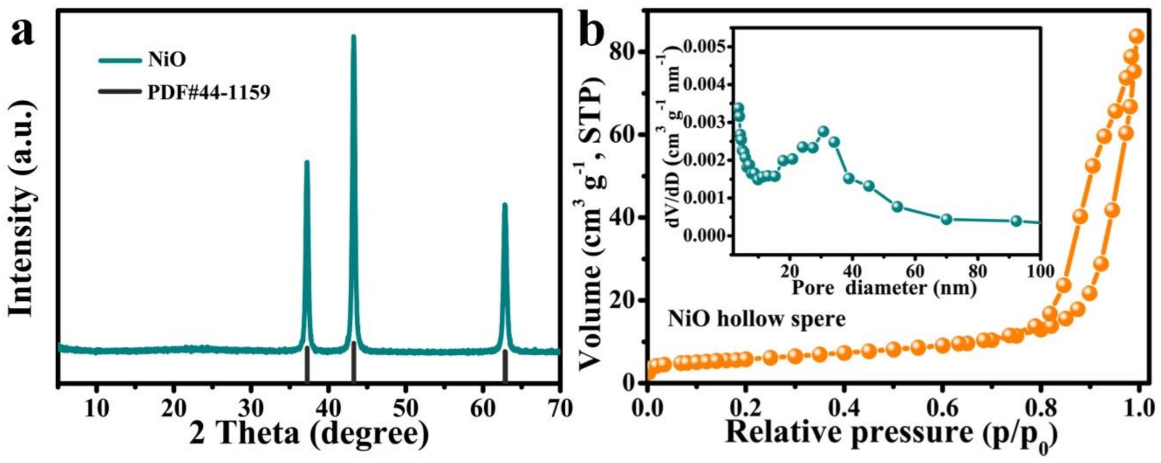

Figure S3. (a) XRD pattern, (b) N_2 adsorption and desorption and pore size distribution curves of NiO hollow spheres

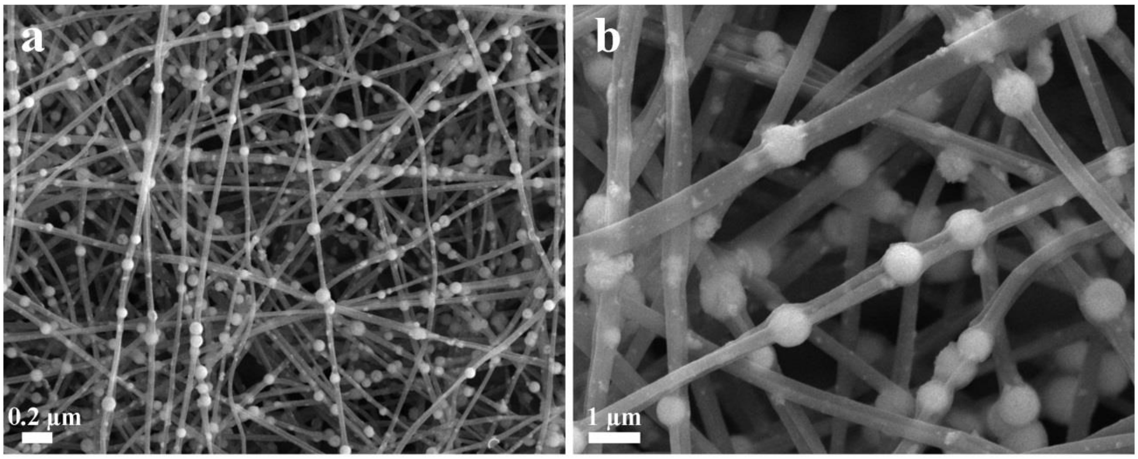

Figure S4. FESEM images of NiO-PAN fibers

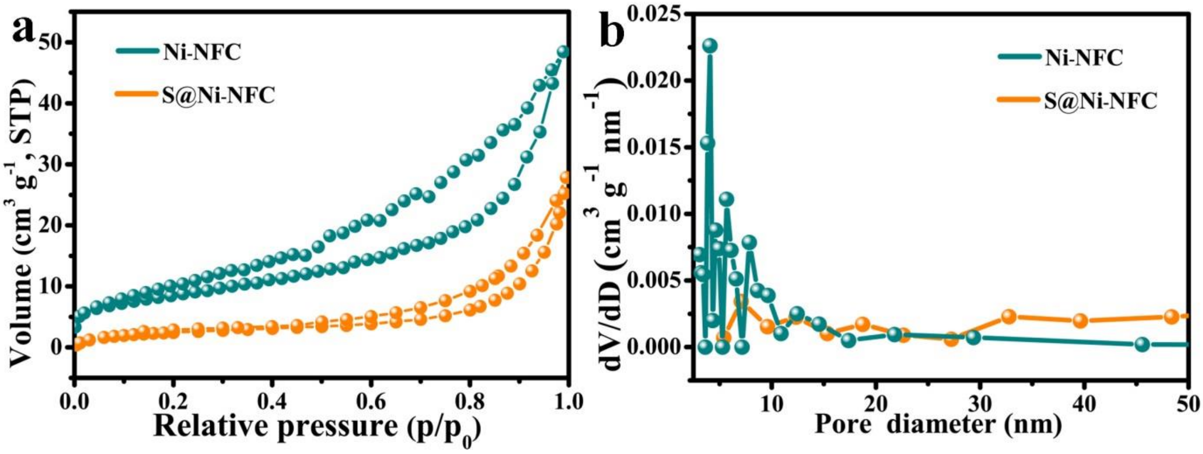

Figure S5. (a) N_2 adsorption/desorption curves and (b) pore size distribution curves of Ni-NCFs and S@Ni-NCFs composites.

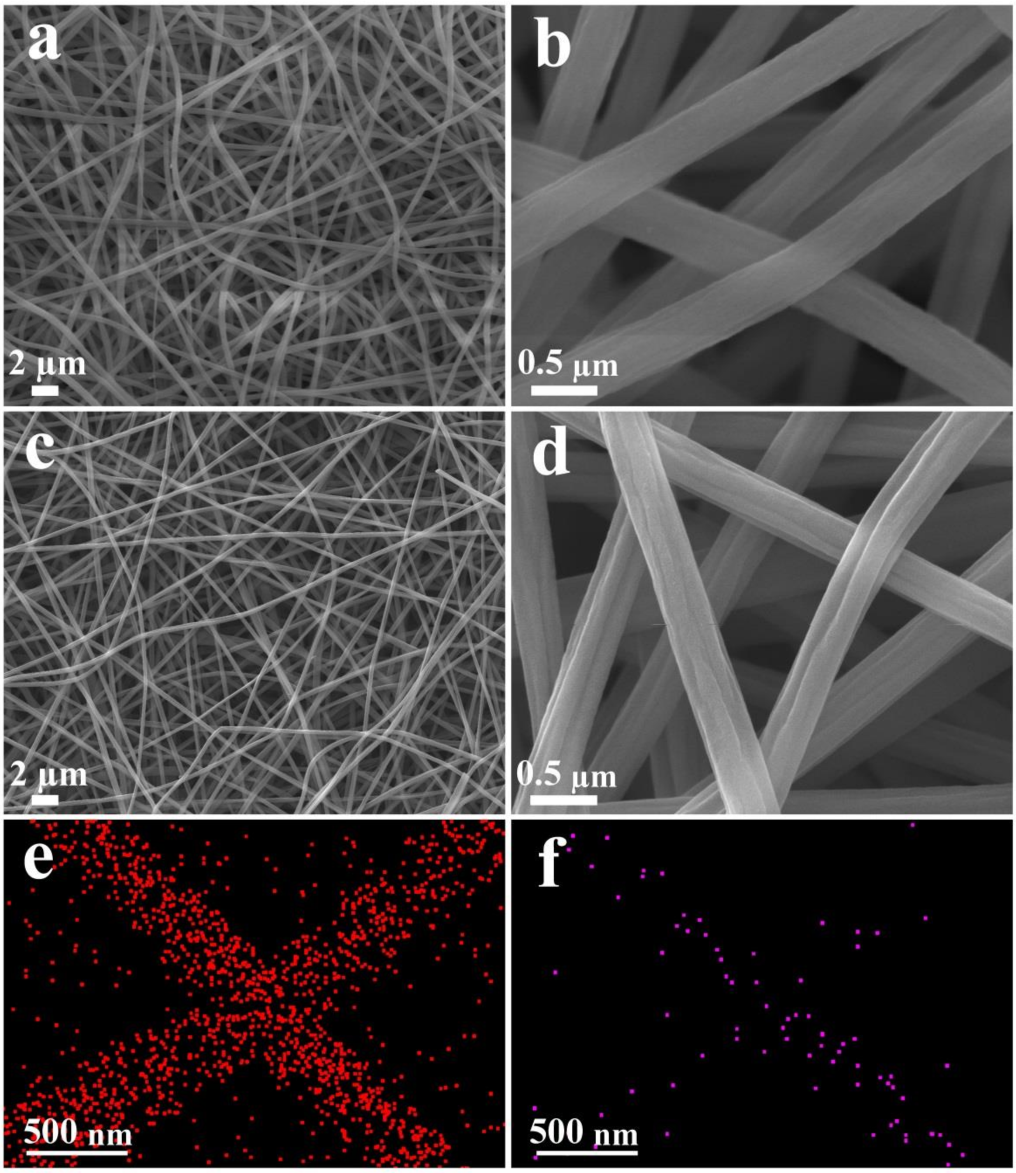

Figure S6. (a-b) FESEM images of PAN and (c-d) NCFs fibers, (e-f) EDS mappings of NCFs

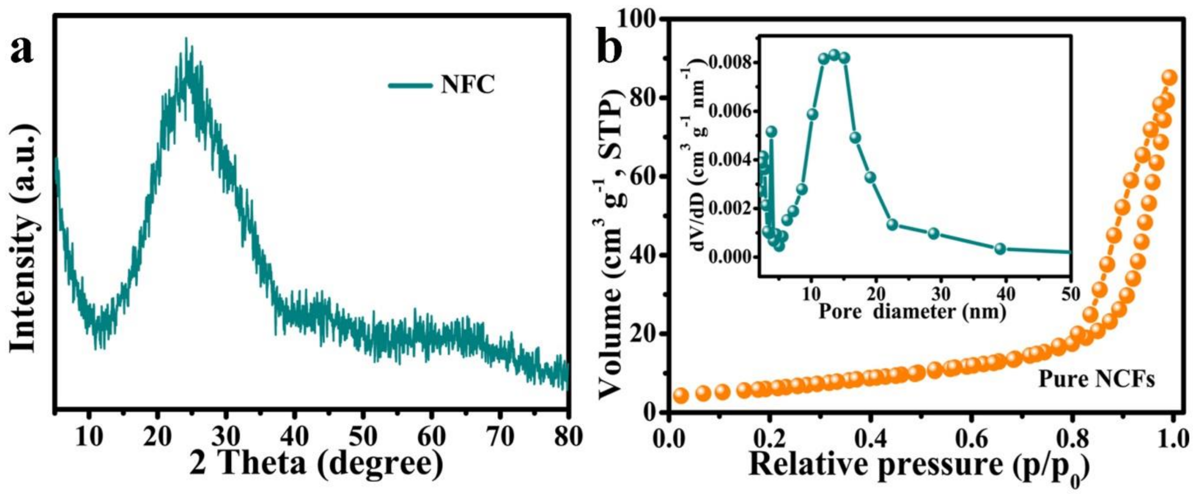

Figure S7. XRD pattern (b) N_2 adsorption and desorption and pore size distribution curves of NCFs fibers

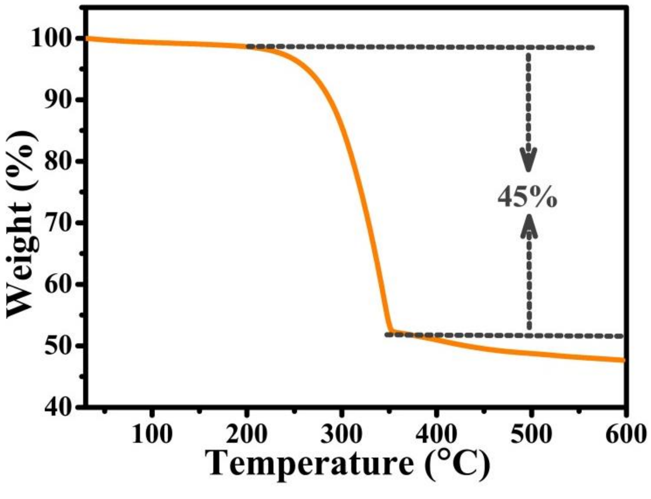

Figure S8 The TGA curve of S@NCFs composite in the nitrogen atmosphere

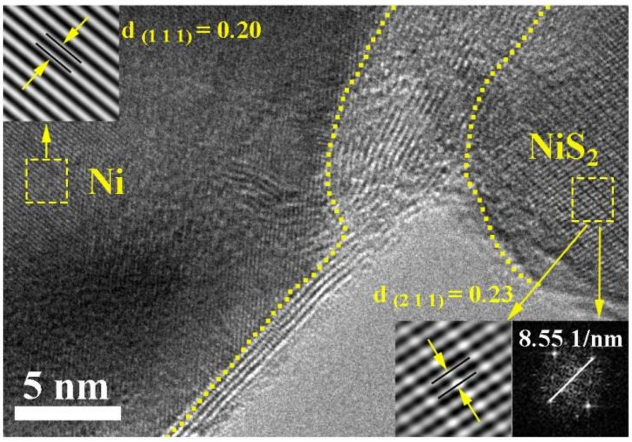

Figure S9 HRTEM image of Ni-NCFs/S composite and the illustrations are lattice fringe and FFT image

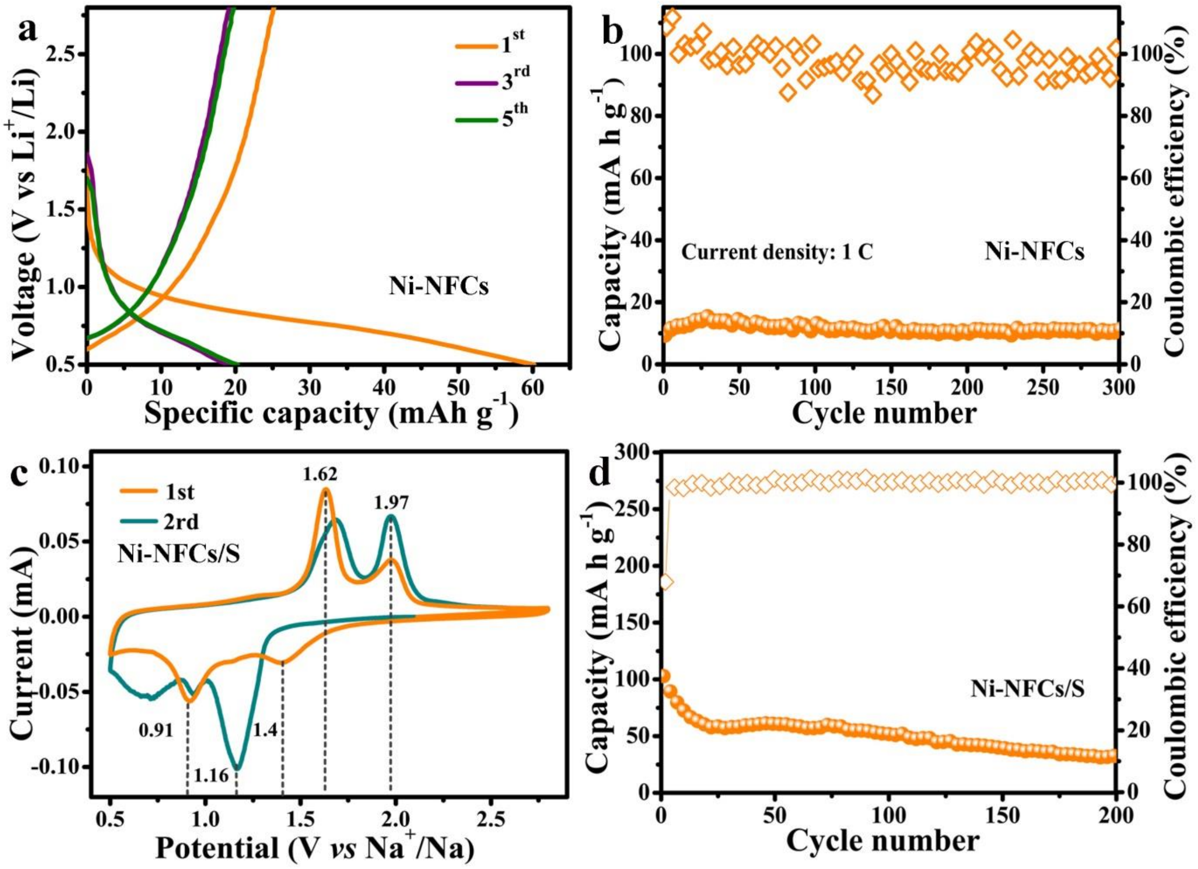

Figure S10 (a) charge/discharge profiles and (b) cycling performance of Ni-NCFs at 1.0 C, (c) CV curve and (d) cycle performance of Ni-NCFs/S electrode at 0.5 C.

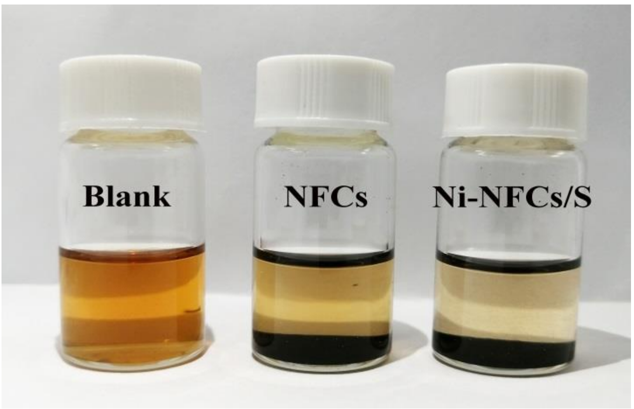

Figure S11. Static adsorption of Na_2S_6 by NCFs and Ni-NCFs/S

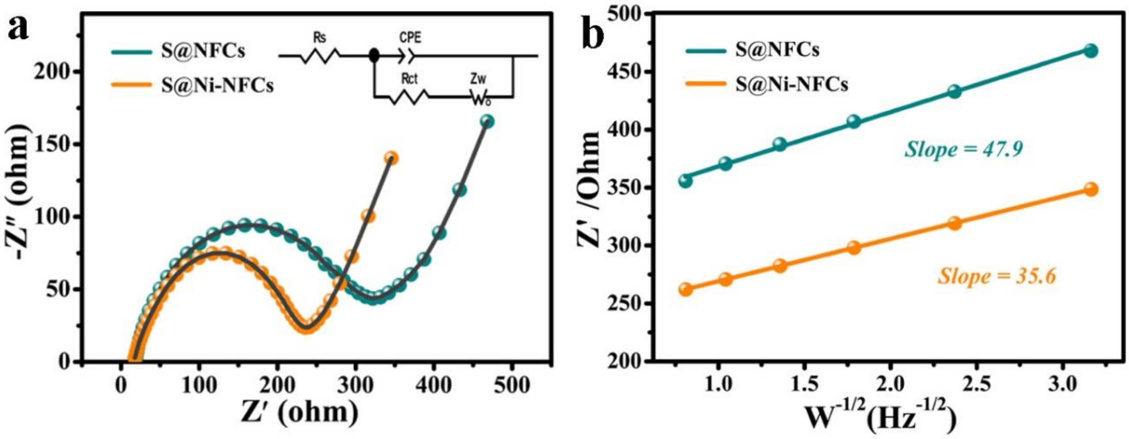

Figure S12. (a) EIS spectra at the open-circuit voltage before initial discharging (inset is the equivalent electrical circuit), (b) reciprocal square root of angular frequency ($\omega^{-1/2}$) dependence of the real impedance (Z′) in the low frequency of S@Ni-NCFs and S@NCFs electrodes

Table S1. Fitting parameters of Na_2S_6 symmetric cells, S@Ni-NCFs and S@NCFs electrodes

**Cell/Sample**	**Rs (Ω)**	**Rct (Ω)**	**Zw (Ω)**
S@NCFs	18.0	261.1	258.9
S@Ni-NCFs	17.66	195.9	59.66
NCFs with Na_2S_6	15	760.6	500.4
Ni-NCFs with Na_2S_6	12.9	145.7	161.4
Ni-NCFs without Na_2S_6	38.9	624.3	740.2

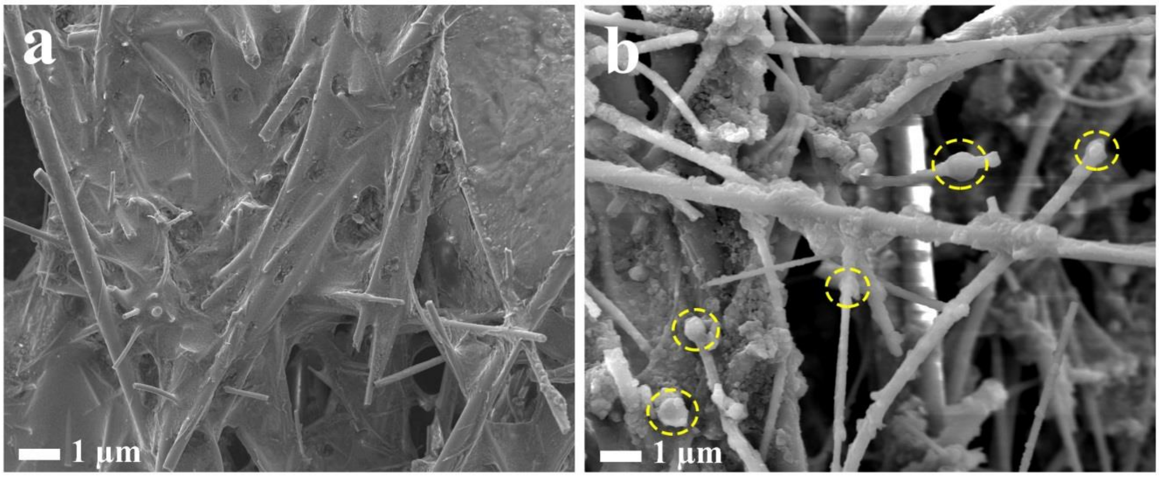

Figure S13. The FESEM images of (a) S@NCFs and (b) S@Ni-NCFs composites after cycling

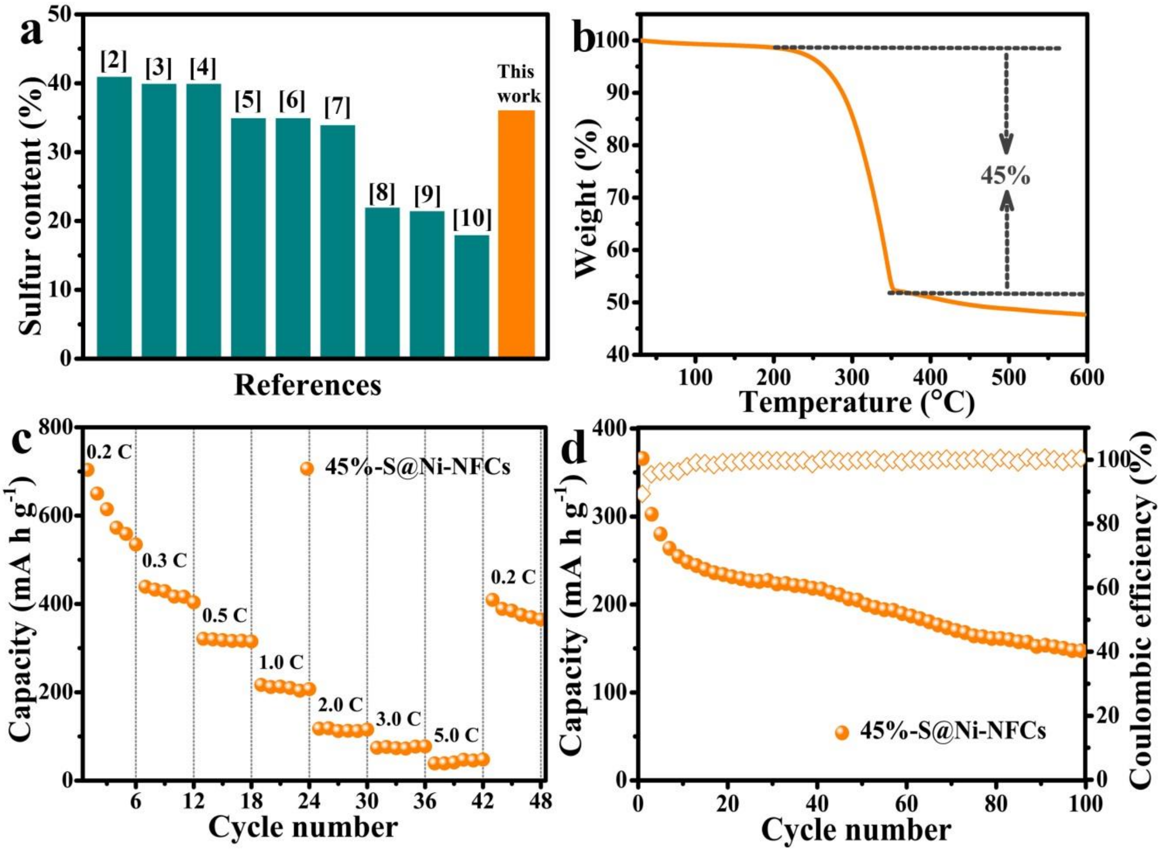

Figure S14 (a**)** Comparison the sulfur content of this work with the literature (b) TGA curve, (c) rate capability and (d) cycling performance of of S@Ni-NCFs with higher sulfur loading.

Table S2. The comparisons of cathode materials for RT Na-S batteries

Samples	S wt.%	Electrolyte	Rate	Cycle number	Decay rate (per cycle)
**S@Ni-NCFs**	**36**	**1 M NaClO4 in TEGDME**	**0.5 C**	**270**	**0.17 %**
PCMs-S [2]	34	1M $NaClO_4$ in PC/EC + 5 wt% FEC	100 mA g^{-1}	350	0.17 %
SPAN[7]	41	1 M $NaPF_6$ in EC/DEC	0.1 C	200	0.11 %
Sulfur infiltrated spheres (S@C) [6]	35	1 M $NaPF_6$ + 0.25M $NaNO_3$ in TEGDME	1 C	1500	0.016 %
CFC/S[11]	24.4	1.5 M $NaClO_4$+ 0.2 M $NaNO_3$ in TEGDME	0.1	300	0.23 %
cZIF-8/S[12]	50 %	1 M $NaClO_4$ in TEGDME	0.2 C	250	0.17 %
CC@MnO_2 /Na_2S_6 [13]	0.5 mg cm^{-2}	1.5 M $NaClO_4$ + 0.2 M $NaNO_3$ in TEGDME	0.2 A g^{-1}	500	0.15 % at 200th 0.023 % at 500th
Thioether bond functionalized carbon (SC)[9]	21.5 %	1 M $NaClO_4$+PC+FEC	100 mA g^{-1}	800	/
S@iMCHS[14]	46 %	1.0 M $NaClO_4$+ PC/EC + 5 wt % FEC	100 mA g^{-1}	200	0.056 %
micoporous carbon/sulfur MCPS[15]	47 %	1 M $NaClO_4$+EC/DEC +SiO_2-IL-ClO_4	0.5 C	100	0.31 %

References

[1] Liu, T., Jia, M., Zhang, Y., Han, J., Li, Y., Bao, S., D. Y. Liu, J. Jiang, M. W. Xu, *J. Power Sources* **2017**, *341*, 53-59.

[2] I. Kim, C. H. Kim, S. Choi, J. P. Ahn, J. H. Ahn, K. W. Kim, E. J. Cairns, H. J. Ahn, *J. Power Sources* **2016**, *307*, 31-37.

[3] W. Y. Du, Y. K. Wu, T. T. Yang, B. S. Guo, D. Y. Liu, S. J. Bao, M. W. Xu, *Chem. Eng. J.* **2020**, *379*, 122359.

[4] B. W. Zhang, T. Sheng, Y. X. Wang, S. Chou, K. Davey, S. X. Dou, S. Z. Qiao, *Angew. Chem.-Int. Edit.* **2019**, *131*, 1498-1502.

[5] T. T. Yang, W. Gao, B. S. Guo, R. M. Zhan, Q. J. Xu, , X. Y. Li, Y. M. Chen, M. W. Xu, *J. Mater. Chem. A* **2019**, *7*, 150-156.

[6] R. Carter, L. Oakes, A. Douglas, N. Muralidharan, A. P. Cohn, C. L. Pint, *Nano Lett.* **2017**, *17*, 1863-1869.

[7] L. Zhang, B. Zhang, Y. Dou, Y. Wang, M. Al-Mamun, X. Hu, H. Liu, *ACS Appl. Mater. Inter.* **2018**, *10*, 20422-20428.

[8] T. H. Hwang, D. S. Jung, J. S. Kim, B. G. Kim, J. W. Choi, *Nano Lett*. **2013**, *13*, 4532-4538.

[9] K. J. Chen, H. J. Li, Y. Xu, K. Liu, H. Li, X. W. Xu, X. Q. Qiu, M. Liu, *Nanoscale* **2019**,*11*, 5967-5973.

[10] L. Fan, R. Ma, Y. Yang, S. Chen, B. Lu, *Nano Energy* **2016**, *28*, 304-310.

[11] Q.-Q. Lu, X.-Y. Wang, J. Cao, C. Chen, K. Chen, Z.-F. Zhao, Z.-Q. Niu, J. Chen, *Energy Storage Mater*. **2017**, *8*, 77.

[12] Y. M. Chen, W. F. Liang, S. Li, F. Zou, S. M. Bhaway, Z. Qiang, M. Gao, B. D. Vogt, Y. Zhu, *J. Mater. Chem. A* **2016**, *4*, 12471-12478.

[13] A. Kumar, A. Ghosh, A. Roy, M. R. Panda, M. Forsyth, D. R. MacFarlane, S. Mitra, *Energy Storage Mater.* **2019**, *20*, 196-202.

[14] Y. -X. Wang, J. P. Yang, W. H. Lai, S. -L. Chou, Q. -F. Gu, H. K. Liu,,D. Y. Zhao, S. X. Dou, J. Am. Soc. **2016**, *138*, 16576-16579.

[15] S. Y. Wei, S. M. Xu, A. Agrawral, S. Choudhury, Y. Y. Lu, Z. Y. Tu, L. A. Archer, *Nat. Commun.* **2016**, *7*, 11722.
